# Neurodegeneration and energy depletion in MS: Links between tissue integrity loss and microvascular changes in white matter disease

**DOI:** 10.1016/j.ynirp.2025.100309

**Published:** 2025-12-10

**Authors:** Linda Sundvall, Mikkelsen Irene Klærke, Brian Hansen, Simon Fristed Eskildsen, Mette Madsen Hjørringgaard, Mikkel Karl Emil Nygaard, Peter Vestergaard Rasmussen, Thor Petersen, Leif Østergaard

**Affiliations:** a Department of Neurology, Aarhus University Hospital, Aarhus, Denmark; b Center of Functionally Integrative Neuroscience (CFIN), Aarhus University, 8000 Aarhus, Denmark; c Section of Neuroradiology, Department of Radiology, Aarhus University Hospital, Aarhus, Denmark; d Neurology Research Unit, University Hospital of Southern Denmark, Aabenraa, Denmark; e Department of Regional Health Research, University of Southern Denmark, Odense, Denmark

**Keywords:** Multiple sclerosis, Diffusion-weighted imaging, Non-Gaussian diffusion MRI measurement, Perfusion-weighted imaging, Capillary transit-time heterogeneity, Biophysical tissue oxygenation estimates

## Abstract

Disturbances in cerebral oxygen delivery and utilization are increasingly recognized as key features, and potential contributors to, neuronal damage in multiple sclerosis (MS). We recently discovered microvascular changes, which are thought to limit oxygen extraction, in MS-related white matter (WM) lesions compared to unspecific WM lesions. It is unclear whether such microvascular changes antedate demyelinating changes in MS, or whether they are secondary to subsequent disease changes, such as inflammation, tissue edema, and blood-brain barrier break-down. Diffusion kurtosis imaging (DKI) is sensitive to early MS-related disease changes, including altered myelin integrity, cellularity, and edema detecting deviations from Gaussian diffusion that serve as indirect markers of tissue integrity. The purpose of this study was to examine whether regions with altered DKI metrics overlap with regions with microvascular changes in MS patients, and to compare microvascular changes with parallel DKI changes in both MS-related lesions and unrelated WM lesions to learn more about their microstructural correlates.

In this cross-sectional study, we assessed microstructural damage in 54 MS patients and 26 non-diseased symptomatic controls (SC) using diffusion kurtosis imaging (DKI) and explored the relationship between these findings and microvascular flow patterns and oxygen delivery measured by dynamic susceptibility contrast-enhanced MRI (DSC-MRI).

MRI at 3T included three-dimensional (3D) T2-weighted fluid-attenuated inversion recovery (T2-FLAIR), 3D magnetization-prepared 2 rapid acquisition gradient-echo (MP2RAGE), post-contrast 3D T1-weighted images, DSC-MRI, and DKI. White matter lesions (WMLs) were manually outlined as MS-characteristic T2-FLAIR lesions, MS contrast-enhancing lesions and nonspecific lesions T2-FLAIR lesions. DKI-derived structural parameters, mean kurtosis (MK) and mean diffusivity (MD), were extracted from lesion masks and normal-appearing white matter (NAWM) and correlated with DSC-derived vascular parameters mean transit time (MTT), and the distribution of capillary transit times (CTH). Finally, an extended flow-diffusion model of oxygen transport was employed to evaluate tissue oxygen availability based on local blood flow and microvascular flow patterns.

After adjusting for age and sex, NAWM in MS showed higher MD (+2.4 %, p = 0.01) and lower MK (−2.8 %, p = 0.01) compared with SC without concurrent changes in perfusion or oxygenation. Unspecific T2-FLAIR lesions demonstrated higher MD relative to NAWM (+13 %, p < 0.001) and reduced MK (−6.7 %, p < 0.001), but no microvascular impairment. By contrast, MS T2-FLAIR lesions showed more pronounced structural alterations, with higher MD than unspecific lesions (+13 %, p = 0.01) and markedly reduced MK (−16 %, p = 0.02), accompanied by increased CTH (+31 %, p = 0.02) and prolonged MTT (+32 %, p = 0.02), consistent with impaired oxygen extraction despite preserved CBF.

Our findings indicate that, microstructural alterations, as assessed by DKI, are detectable in normal-appearing tissue before microvascular disturbances become evident on GE-DSC MRI. In MS lesions, however, microvascular flow heterogeneity coexists with tissue degeneration, suggesting inefficient oxygen extraction as a likely contributor to lesion pathology. These results emphasize the need for longitudinal studies to determine the temporal relationship between impaired oxygen extraction and disease progression.

## Highlights

• Explorative study of capillary flow and energy deficits in MS-related degeneration.
• Cross-sectional imaging of tissue structure, capillary flow, and oxygen delivery.
• Cellular changes, but no vascular issues, in normal-appearing MS brain regions.
• Tissue integrity loss in MS lesions links to impaired capillary oxygen extraction.
• Longitudinal studies needed to clarify microvascular impact on MS progression.

## 1 Introduction

Multiple Sclerosis (MS) is a chronic inflammatory disease characterized by the appearance of focal demyelinating lesions and gradual, diffuse brain damage caused by neurodegenerative processes (Lassmann, 2018). Understanding the mechanisms underlying the chronic inflammation in MS is of significant importance. The resultant brain damage leads to increased disability and a progressive stage, where current treatments show limited efficacy (Cree et al., 2021; Amezcua, 2022). Cerebral energy deficiency, encompassing oxygen-, glucose-, and lipid-related processes that contribute to cellular energy imbalance, is increasingly recognized as a key feature of MS and a potential therapeutic target (Park and Choi, 2020; Kornberg et al., 2018). However, the underlying cause of this energy crisis remains unclear. Possible explanations include altered blood flow (Haider et al., 2016; Wuerfel et al., 2004; Holland et al., 2012; Geraldes et al., 2017), inefficient oxygen extraction from the vasculature into brain tissue, or impaired mitochondrial oxygen utilization within axons and glia (Aboul-Enein et al., 2003; Trapp and Stys, 2009; Witte et al., 2014; Chandler et al., 2023; Mahad et al., 2015).

In recent work, we employed dynamic susceptibility contrast-enhanced MRI (DSC-MRI) to assess the distribution of oxygenated blood and discovered significant microvascular changes, which are thought to restrict efficient oxygen extraction, in MS-related white matter (WM) lesions compared to nonspecific WM lesions. Reactive oxygen species production, oxidative damage (Trapp and Stys, 2009; Mahad et al., 2015; Haider et al., 2011; Lu et al., 2000), and impaired cerebrovascular reactivity (Vestergaard et al., 2022) have also been implicated in MS, and provide further evidence of underlying vascular alterations, such as reduced vascular compliance and a disturbed balance between neural demand and oxygen delivery, particularly during the progressive stage.

Studies demonstrate that oxygen utilization is indeed reduced in MS patients (Chandler et al., 2023; Ge et al., 2012), even in the absence of cerebral atrophy and cerebral blood flow (CBF) changes (Knudsen et al., 2024). Given that total oxygen consumption equals CBF multiplied by the local oxygen extraction (OEF), these findings reflect either compromised oxygen extraction, reduced metabolic demand due to cellular dysfunction, or both. The interplay between hypoxia and inflammation in MS is especially intriguing, as hypoxia may worsen inflammation and promote additional tissue damage (Haider et al., 2016; Mahad et al., 2015).

Efficient oxygen extraction relies on an even distribution of oxygenated blood across capillaries (Stefanovic et al., 2008), and may therefore be compromised by “shunting” of oxygenated blood caused by severe disruptions in capillary flow (Østergaard et al., 2013, 2015; Jespersen and Østergaard, 2012; Angleys et al., 2015). DSC-MRI, combined with a biophysical model of oxygen transport, now allow for the identification of tissues with impaired capillary flow and oxygen delivery (Jespersen and Østergaard, 2012; Mouridsen et al., 2014). Whether these microvascular changes precede or follow demyelinating processes in MS remains unclear, raising questions about their role as either early disease drivers or secondary effects of inflammation and neurodegeneration.

Diffusion-weighted MRI is sensitive to water molecules’ micrometer-scale Brownian motions, and quantification of the directionality of water diffusion by diffusion tensor imaging (DTI) has proven informative in assessing structural brain damage in MS(26, 27). Diffusion kurtosis imaging (DKI), an extension of DTI, provides a more comprehensive characterization of water diffusion, offering indirect markers of microstructural complexity and demonstrating significant utility in investigating structural changes in MS (Bester et al., 2015; Thaler et al., 2021; de Kouchkovsky et al., 2016; Yoshida et al., 2013; Li et al., 2018; Luo et al., 2024; Zhu et al., 2022) and other neurological disorders (Li et al., 2022; Wang et al., 2022; Gao et al., 2024; Hu et al., 2022).

DKI achieves its sensitivity to tissue microstructure by quantifying deviations from free (Gaussian) diffusion of water in tissue. Unlike DTI, which assumes Gaussian diffusion, DKI is sensitive to water molecules’ interactions with cell membranes and organelles, and transport among biological compartments, all of which contribute to deviations from free diffusion. Because these deviations are linked to tissue microstructure, DKI provides sensitive, indirect indices of microstructural integrity, with large, positive kurtosis values indicating greater tissue complexity and heterogeneity (Maiter et al., 2021). In rodent models of demyelination, DKI metrics have shown strong correlations with histological outcomes (Guglielmetti et al., 2016; Falangola et al., 2014; Chuhutin et al., 2020; Kelm et al., 2016). In MS, neurodegeneration and demyelination typically result in higher mean diffusion (MD) and lower mean kurtosis (MK) compared to healthy tissue (Guglielmetti et al., 2016; Falangola et al., 2014). Demyelination increases diffusion in directions perpendicular (radial diffusivity, RD) or parallel to nerve fibers (axial diffusivity, AD) (Guglielmetti et al., 2016; Falangola et al., 2014), and AD alterations have been linked to axonal degeneration (Sun et al., 2006), microgliosis (Guglielmetti et al., 2016), and astrogliosis (Guglielmetti et al., 2016; Falangola et al., 2014; Wang et al., 2009). Similarly, reductions in radial kurtosis (RK) and axial kurtosis (AK) are known to occur in cases of chronic demyelination (Falangola et al., 2014). Additionally, decreases in the DKI-derived axonal water fraction (AWF, a white matter metric that measures how much of the MR-visible water resides inside the axons) is associated with reduced axonal density (Guglielmetti et al., 2016) (29). DKI-derived indices have demonstrated sensitivity to tissue abnormalities in both early and progressive stages of MS (van der Weijden et al., 2022; Thaler et al., 2021; Zhu et al., 2022; Riemenschneider et al., 2022), correlating with clinical progression, disability (de Kouchkovsky et al., 2016; Chuhutin et al., 2020; Spampinato et al., 2017; Zhu et al., 2023), and cognitive decline (Bester et al., 2015; Nygaard et al., 2021).

In this cross-sectional study, we assessed microstructural damage in 54 MS patients and 26 non-diseased controls using DKI. We explored its relationship with microvascular flow patterns and oxygen delivery, as measured by DSC-MRI. Specifically, we aimed to examine whether tissue integrity loss in MS is associated with alterations in microvascular perfusion and whether such relationships extend beyond demyelinating MS lesions into normal-appearing brain regions.

Furthermore, we evaluated whether DKI and DSC-MRI can distinguish MS-specific demyelinating lesions from non-specific white matter hyperintensities, frequently observed as incidental findings on T2-weigthed images, which often prompt diagnostic uncertainty and additional follow-up imaging (Kaisey et al., 2019; Solomon et al., 2016; Solomon and Weinshenker, 2013; Filippi et al., 2019).The DSC data were also part of the findings reported by Sundvall et al. (2024)

## 2 Methods

### 2.1 Materials

A consecutive cohort of patients referred for suspected MS was recruited upon admission to the MS clinic from January 2018 to June 2020. Inclusion criteria comprised patients aged >18 years who had not undergone disease-modifying therapy or received immunosuppressive drugs. Patients were excluded in case of concurrent morbidity (autoimmune disease, other neurologic disease, active cancer), an acute inflammatory process (leukocyte count >11,000/ml), or contraindications to contrast media injection (eGFR <45 ml/min). A subgroup of patients in this study were included in our DSC study conducted at the same time. All subjects provided informed consent for clinical examination and central nervous system (CNS) MRI examination.

### 2.2 Clinical data registration

Subjects were allocated into either the MS group or a cohort referred to as non-diseased Symptomatic Controls (SC). Diagnosis of MS and disease courses were conducted in accordance with the 2017 McDonald criteria (Thompson et al., 2018). SCs are individuals referred to the Department of Neurology with neurological symptoms raising suspicion of MS, but whose symptoms were not considered typical of MS or other demyelinating diseases after comprehensive evaluation. All SCs had normal findings on neurological examination, blood tests, CNS MRI, and neurophysiological testing when indicated, and were classified as SC based on exclusion criteria. Previous studies have indicated SC does not represent early or subclinical MS (Teunissen et al., 2013). Clinical data were collected during the subjects' initial visit and at the first follow-up approximately six months later. The recorded clinical characteristics encompassed the Expanded Disability Status Scale (EDSS) and disease course (Clinical Isolated Syndrome (CIS), Radiologic Isolated Syndrome (RIS), Relapsing-Remitting MS (RRMS), Primary-Progressive MS (PPMS)). Patients referred due to incidental MRI findings suggestive of MS, but without typical MS symptoms, were diagnosed with Radiologically Isolated Syndrome (RIS). We conducted a diagnostic verification of all patient records at least two years after recruitment.

### Ethical approval

Written informed consent was obtained from all patients before inclusion. The Central Denmark Region Committee on Research Ethics (no. 1-10-72-180-17) and the Danish Data Protection Agency (no. 2012-58-006) approved the study. The study was registered at ClinicalTrials.gov (no. NCT03906370).

### 2.3 MRI protocol

The MRI protocol included three-dimensional (3D) T2-weighted fluid-attenuated inversion recovery (T2-FLAIR; 1.0 mm^3^ isotropic voxels, TR/TE/TI ≈ 5000/388/1800 ms) images and post-contrast 3D T1-weighted (T1 1.0 mm^3^ isotropic voxels, TR/TE/TI ≈ 1900/2.5/900 ms) images of the brain and the spinal cord, for clinical diagnosis and *lesion segmentation* purposes. For research analyses, we additionally acquired pre-contrast 3D T1 magnetization-prepared 2 rapid gradient-echo (MP2RAGE 0.9 mm^3^ isotropic voxels, TR ≈ 6500 ms, dual inversion times 500/2900 ms) images for *tissue segmentation*, a diffusion kurtosis imaging (DKI) sequence (2.0 mm^3^ isotropic voxels, TR/TE ≈ 2260/63 ms, b-values 0–2500 s/mm^2^ across 201 directions) for assessment of *tissue microstructure*, and a dynamic susceptibility contrast (DSC) sequence (3.0 mm^3^ isotropic voxels, TR/TE ≈ 800/32 ms, flip angle 45–53°, 42 slices) for *microvascular perfusion and biophysical tissue oxygenation estimates*. DSC scans were acquired with gadolinium contrast (0.1 mmol/kg at 5 ml/s, followed by 30 ml saline flush. All images were acquired on a Siemens Magnetom Prisma 3T scanner.

#### 2.3.1 DKI data processing and parameter estimation

Briefly, DKI data were denoised (Veraart et al., 2016), Gibbs-corrected (Kellner et al., 2016), and eddy-current and motion corrected using standard tools from the FMRIB Software Library (FSL) (Andersson and Sotiropoulos, 2016). The standard DKI parameters used here were estimated using in-house MATLAB (MathWorks, USA) scripts. (see Supplementary Methods for details). The investigated metrics included mean diffusivity (MD, × 10^−3^ mm^2^/s), reflecting the overall magnitude of water diffusion; axial diffusivity (AD, × 10^−3^ mm^2^/s), representing diffusion along axons; radial diffusivity (RD, × 10^−3^ mm^2^/s), representing diffusion perpendicular to axons; mean kurtosis (MK, unitless), capturing overall microstructural complexity; radial kurtosis (RK, unitless), describing kurtosis perpendicular to axons; and axonal water fraction (AWF, unitless), estimating the proportion of intra-axonal water relative to total water content.

#### 2.3.2 Investigation of *impaired microvascular flow* patterns and oxygen delivery (DSC)

We employed a methodology developed at the Centre of Functional Integrative Neuroscience (CFIN) at Aarhus University to determine hemodynamic parameters (Mouridsen et al., 2014) and to derive the resulting tissue oxygenation using an extended version of the classic flow-diffusion equation (22). The CFIN DSC-processing pipeline was developed in MATLAB (MathWorks USA). The resulting DSC metrics are listed in Table 1. Briefly, employing an algorithm specifically designed for diseases with anticipated contrast agent extravasation (Hansen et al., 2017), our DSC measurements produced leakage-insensitive maps of cerebral perfusion parameters: CBF, cerebral blood volume (CBV), mean transit time (MTT), and the distribution of capillary transit times (CTH). While MTT is the mean red blood cell transit time through the capillary bed, CTH represents the standard deviation of this distribution (Jespersen and Østergaard, 2012). The distribution was modeled with a family of gamma variate functions (Mouridsen et al., 2014). Finally, combining CTH and MTT, the extended flow-diffusion model (Jespersen and Østergaard, 2012) estimates tissue oxygen tension estimation (P_t_O_2_), assuming that net oxygen extraction meets the net demand of resting brain (CMRO_2_ max = 2.5 ml/100 ml/min).

**Table 1:** Investigated DSC metrics.

	Biophysical measurement	Biophysical interpretation
rCBV	The CBV relative to the average CBV in deep NAWM.	rCBV values reflect microvascular density and vasodilator responses
rCBF	The CBF relative to the average CBF in deep NAWM	Brain oxygen metabolism equals CBF multiplied by the oxygen extraction fraction from the vasculature. Capillary flow disturbances limit oxygen extraction and are therefore expected to affect the CBF needed to meet local energy demand.
MTT	Mean of the distribution of capillary transit times.	MTT equals the CBV: CBF ratio. During hypoperfusion, reduced CBF therefore prolongs MTT, allowing more time for blood-tissue oxygen diffusion exchange before blood returns to the heart.
CTH	Capillary transit time heterogeneity. The standard deviation of the distribution of capillary transit times.	Heterogeneous microvascular flow causes “shunting” of oxygenated blood through capillaries with short transit times. During hyperemia, capillary flows homogenize, maintaining high OEF(22). Conditions with capillary flow disturbances are characterized by elevated CTH, and an inability to homogenize capillary flow patterns to satisfy energy demands
PtO_2_	The tissue oxygenation that would result, if tissue where to support the minimum oxygen requirement of resting brain, given the measured CTH and MTT values	The original flow-diffusion equation takes CBF, CBV, and MTT into account, but not blood's capillary distribution into account. The extended flow-diffusion equation includes CTH and relates, MTT, CTH, and CBF to CMRO_2_ for a given P_t_O_2_ level at steady state – and *vice versa*.

Due to technical and physiological variations in absolute MRI DSC perfusion measures, CBF and CBV estimates are not reliably comparable across individuals. CBF and CBV were normalized to deep normal-appearing white matter (NAWM) ROI within each subject to account for interindividual variability.

#### 2.3.3 Lesion mask delineation

Binary white matter lesion (WML) masks were outlined on sagittal 3D T2-FLAIR images and post-contrast sagittal 3D T1 images. Employing intensity thresholding and level sets, multiple masks were manually delineated on each image slice to encompass lesion areas categorized as:

• MS T2-FLAIR LESIONS: WML hyperintense on T2-FLAIR but not on post-contrast T1 images, with the radiological features characteristic of MS
• MS T1 LESIONS: WML hyperintense on post-contrast T1 and T2-FLAIR, with the radiological features characteristic of MS
• UNSPECIFIC T2-FLAIR LESIONS: Unspecific T2-FLAIR lesions were defined as small, non-confluent white matter hyperintensities lacking the radiological features characteristic of MS lesions (e.g., ovoid shape, periventricular orientation, Dawson's fingers, juxtacortical location) and without radiological characteristics indicative of infectious, inflammatory, neoplastic, microhemorrhages, small-vessel ischemic, or large ischemic etiologies. In SC individuals, such lesions were interpreted as unrelated to demyelinating disease, consistent with their normal clinical and radiological findings and their stable 2-year follow-up without neurological disease. In MS patients, such lesions were only included when radiological appearance clearly deviated from typical MS lesions, as determined and confirmed by a neuroradiologist.

Lesion delineation excluded signals ascribed to motion artifacts, ventricular edge effects, skull, or signal inhomogeneities, and were limited to white matter lesions and cortical lesions within the cerebral hemispheres with a minimum size of 3 voxels (3.0 × 3.0 mm). Disease MS lesions vary significantly in size, from a few millimeters to several centimeters. Both lesion size and the MRI sequence's spatial resolution influence the contribution of surrounding tissue to the signal intensity within lesion ROIs. To mitigate partial volume effects (PVE), we required lesions to have a cross-section of at least 3 mm (3 pixels) in at least two contiguous slices on T2-FLAIR images during manual segmentation. Additionally, DKI voxels (2 mm in-plane voxel size) and DSC voxels (3 mm in-plane voxel size) that contained less than 50 % of the lesion, as determined by the higher-resolution segmentation and nearest neighbor analyses, were excluded from the DKI and DSC parameter analyses. Lesion masks were manually drawn by an experienced operator (LS, one year-of-experience) and validated by a certified neuroradiologist (MH, ten years-of-experience) for accuracy and differential diagnosis. An automatically generated WML mask was created using the MATLAB-based lesion segmentation toolbox (LST version toolbox 2.0.15) (www.statisticalmodelling.de/lst.html) implemented in Statistical Parametric Mapping toolbox (SPM12) running in MATLAB R2016b (MathWorks, Natick, MA, USA) and combined with the manually drawn lesion mask. Together, the auto-generated WML mask and the manually drawn lesion masks served as an exclusion mask when generating NAWM and normal-appearing gray matter (NAGM) masks from tissue segments.

#### 2.3.4 Tissue segmentation

Prior to tissue segmentation, the high-resolution T1-weighted images (MP2RAGE images) were denoised and skull-stripping applied to remove non-brain tissues. After co-registration to Montreal Neurological Institute (MNI) space, brain tissue was segmented into specific types (e.g., GM, WM, and CSF) and voxels labelled according to specific anatomical structures, including the thalamus, cortex, and corpus callosum. Furthermore, manually defined frontal and parietal deep WM regions were delineated in each hemisphere and registered to each subject's T1 MP2RAGE image. This mask was also utilized for the normalization of DSC parameters (Table 2). The automatically generated WML mask, combined with the manually drawn WML mask, served as exclusion masks in the NAWM and NAGM segmentation. Regional volumes were calculated and normalized to segmented whole brain volume (i.e. sum of the GM and WM volumes, including cerebellum and brainstem). Supplementary methods for specifics on the postprocessing protocol.

**Table 2:** Clinical, demographic and tissue volume characteristic of MS and SC.

Parameters	n_MS_/n_SC_	MS	SC	p-value
No of patient (n)		54	26	
Age mean (sd)		38 (11)	36 (13)	0.34
Sex				0.62
Female		61 % (33)	69 % (18)	
Neurological complaints				0.39
Opticus neuritis symptoms^a^ %(n)		30 % (16)	12 % (3)	
Sensory^b^ %(n)		50 % (27)	65 % (17)	
Motor^c^ %(n)		13 % (7)	15 % (4)	
Cerebellar^d^ %(n)		6 % (3)	8 % (2)	
Other^e^		2 % (1)	0 % (0)	
Time since last relapse (months)		6.5 (21)	–	
EDSS mean (sd)	53/0	1.5 (1.5)	NA	
Disease stage phenotype %(n)				
CIS		15 % (8)	NA	
RIS		13 % (7)	NA	
RRMS		59 % (32)	NA	
PPMS		13 % (7)	NA	
Total brain volume mean (sd) cm^3^		1408.41 (123.01)	1409.88 (134.31)	0.96
Thalamus^f^ mean (sd) cm^3^		11.42 (1.85)	12.33(1.30)	0.03
Normalized Thalamus volume^g^		8.1e-03 (1.3e-03)	8.8e-03 (6.8e-04)	0.03
Cortex mean (sd) cm^3^		595.0(56.57)	602.21(63.81)	0.61
Normalized Cortical volume^g^		0.42 (0.03)	0.43 (0.02)	0.35
Unspecific T2-FLAIR lesion^h^ mean (sd) cm^2^	34/8	0.07 (0.07)	0.26 (0.24)	<0.001
Normalized volume^g^		5.6e-05 (4.8e-05)	2.3e-04 (1.7e-04)	<0.001
Enhancing T1-lesion^h^ mean (sd) (cm^2^)	16/0	0.44 (0.96)	0	
Normalized volume^g^		3.3e-04 (8.0e-04)		
MS T2-FLAIR lesion^h^ mean (sd) cm^2^	48/0	2.43 (3.84)	0	
Normalized volume^g^		1.7e-03 (2.7e-03)		

Note: a: poor color vision, vision loss, eye pain. b: numbness, pain, tingling, burning. c: gait change, spasticity. d: balance or coordination problems, slurred speech, vertigo. e: patient with RIS. f: The sum of left and right thalamus. g: Normalized to segmented whole brain volume. h: Lesion load based on manually outlined lesion masks co-registered to DKI & DSC maps.

#### 2.3.5 Region of Interest analyses

Anatomical T2-FLAIR, MP2RAGE, and T1-images were co-registered and resliced using SPM12 to both DKI (b = 0) and DSC (mean image), and the transformations were applied to all masks. DKI-derived and DSC-derived metrics were then extracted from manually drawn WML masks and NAWM and NAGM compartments to conduct a Region of Interest (ROI) analysis for each subject. In ROI analysis for NAWM/NAGM compartments, the WM/GM mask was used as the inclusion and auto-generated WML masks, and the manually drawn WML masks were used as exclusion masks. See Supplementary Methods for further details. Briefly, MK and MD were computed for all ROIs, while values observed along diffusion directions (AD, RD, RK) were computed specifically for WM compartments. The following ROIs were investigated: Lesion masks (Unspecific T2-FLAIR lesions, MS T2-FLAIR lesions, and MS T1lesions), Corpus callosum NAWM, Cortical NAGM, Thalamus NAGM, and deep NAWM.

Fig. 1 ROI placement examples on T1-and T2-weighted images, and DKI parametric maps.

**Fig. 1:**
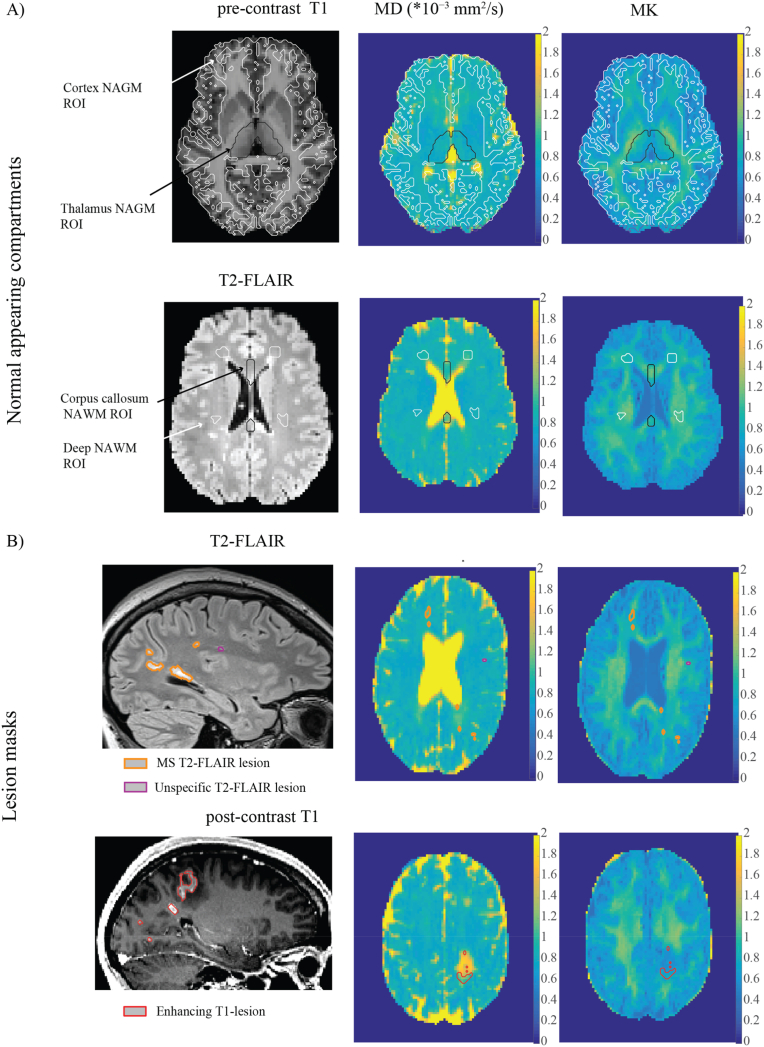
Examples of Region of Interest (ROI) placement on T1-and T2-weighted images and corresponding DKI parametric maps. Example of NAGM and NAWM ROIs on co-registered T2-FLAIR images and pre-contrast T1-images, and as overlays on MD and MK parametric maps, after co-registration and re-slicing to DKI. B) Example of lesion mask on original T2-FLAIR images and post-contrast T1-images, and as overlays on MD and MK parametric maps, after co-registration and re-slicing to the corresponding axial imaging slice in DKI.

### 2.4 Statistics

Statistical analyses were conducted using STATA 17.0 (StataCorp LLC, Texas, USA).

We hypothesized that regions showing significant alterations in microvascular perfusion and oxygen delivery (as measured by DSC-MRI) would correspond to disease-affected areas exhibiting abnormal diffusion metrics (as measured by DKI). Furthermore, we hypothesized that DKI and DSC-MRI signatures measured within outlined MS-specific T2-FLAIR lesions would differ from those measured in non-specific white matter hyperintensities (Unspecific T2-FLAIR lesions).

Normality assumptions were assessed through histograms and QQ plots. Continuous data were presented as mean ± SD or median (interquartile range, IQR) and analyzed using paired t-tests or Wilcoxon signed-rank tests for normally and non-normally distributed data, respectively. Spearman's rank correlation coefficient was interpreted as no correlation at all (=0), weak (≥0.1 and < 0.4), moderate (≥0.4 and < 0.7), strong (≥0.7 and < 1), and perfect (=1). Potential confounding factors that may affect brain diffusivity (age and sex), were included in multiple linear- and logistic regression models for assessing DKI metric biomarker utility. To reduce individual scaling effects, all volume measurements were normalized to the segmentation-based whole brain volume. Power calculations were performed to determine the required sample size for detecting differences between two groups, using MD in corpus callosum NAWM as the primary endpoint. To achieve 80 % power at a significance level of 0.05, it was estimated that 22 participants per group would be necessary. A two-sided P-value of <0.05 was considered significant throughout. As this is an exploratory study, no formal multiple comparison correction was applied. Instead, we focused on identifying converging patterns across modalities and tissue compartments, since vascular impairment is evaluated from a panel of related measures (e.g., CBF, CBV, MTT, CTH, PtO_2_), and strict corrections assuming independence could obscure biologically meaningful effects.

## 3 Results

During the study period, 93 patients were screened, with 80 meeting the inclusion criteria. The DKI dataset comprised 54 MS subjects (ranging from subclinical disease stages (RIS) over CIS and RRMS to progressive MS) and 26 SC, with ROI analysis performed for NAWM and NAGM compartments. Among the MS group, 50 individuals had MS T2-FLAIR lesions, 17 had enhancing T1-lesions, and 34 had unspecific T2-FLAIR lesions. Two individuals had MS T2-FLAIR lesions, which were too small for analysis (see 2.4.3 Lesion mask delineation). Unspecific T2-FLAIR lesions were observed in 8 SC subjects. ROI analysis was conducted for T2-FLAIR lesions in 48 MS subjects and for enhancing T1-lesions in 16 MS subjects. Additionally, ROI analysis for unspecific T2-FLAIR lesions was performed in 8 SC and 34 MS subjects. By definition, SC individuals had no radiological characteristics of MS, and their T2-FLAIR hyperintensities were classified exclusively as unspecific. The integrated DKI-DSC dataset was derived from 50 MS subjects and 8 SC who underwent the full DKI and DSC imaging protocols. Supplementary Material Fig. S1 provides and overview of the patient recruitment.

### 3.1 Clinical, demographic and tissue volume characteristic

The MS patients and SC cohorts exhibited comparable ages (38 vs. 36 years) and equal gender distribution (female 61 % vs 69 %). Most MS patients were characterized by mild disability (mean EDSS 1.5) and disease-stage RRMS (59 %) (Table 3). MS patients exhibited a higher prevalence of oligoclonal bands in the CSF (74 vs 8 %) and a higher prevalence of elevated Immunoglobulin G index (54 vs 8 %) compared to SC. Although SC came to clinical attention in the same way as the MS cohort, all SC maintained their non-diseased status over a 2-year period. The conditions encountered among SC included paresthesia without sensory change, muscle tension, visual adjustment issues, migraines, anxiety disorders. There was no significant difference in blood hemoglobin content between MS and SC (8.7 vs 8.8 mmol/L). Two SC participants showed positive CSF oligoclonal bands but had no evidence of neurological disease after thorough evaluation and remained without diagnosis during 2 years of follow-up.

**Table 3:** WM lesion's microstructural and microvascular characteristics.

	MD	MK	rCBF	CTH	MTT	PtO_2_
MS NAWM vs SC NAWM						
	↑	↓	∼	∼	∼	∼
MS T2-lesion vs SC Unspecific T2-lesion						
	↑	↓	∼	↑	↑	↓
MS T2-lesion vs NAWM						
	↑	↓	↑	↑	↑	↓
MS Unspecific T2-lesion vs NAWM						
	↑	↓	∼	↓	↓	↑
SC Unspecific T2-lesion vs NAWM						
	↑	↓	∼	↓	↓	↑

MS T2-FLAIR and unspecific lesions showed differences in both anatomical distribution and volume. MS lesions were mainly periventricular and frequently involved the corpus callosum, whereas unspecific lesions were predominantly subcortical, though occasionally periventricular. Unspecific lesions were also considerably smaller than MS lesions (Table 2). There was no significant difference in the normalized volumes of any of the WM compartments, but MS patients showed a significantly lower segmented Thalamus volume compared to SC. Paraclinical characteristics including cardiovascular comorbidities are shown in Supplementary materials Table S1.

### 3.2 Changes in microstructural and microvascular parameters across regions

DKI-derived MD was significantly higher in NAGM regions (0.99 ± 0.05 × 10^−3^ mm^2^/s) compared to NAWM regions (0.96 ± 0.05 × 10^−3^ mm^2^/s). MK in NAGM (0.88 ± 0.17) was significantly lower than in NAWM (1.04 ± 0.04; p < 0.05). Diffusion was predominantly axial in NAWM while predominantly radial in MS T2-FLAIR lesions. Within NAWM, the corpus callosum exhibited significantly higher RK (1.65 ± 0.13) compared to frontal and parietal deep NAWM (1.31 ± 0.07; p < 0.05), consistent with the influence of WM geometry and axonal alignment on DKI metrics.

DSC-derived flow patterns varied by region, showing an inverse relation between perfusion and CTH as expected. Higher rCBF and shorter MTT were observed in highly vascularized NAGM compared to deep NAWM, with a tendency toward higher CTH in deep NAWM compared to NAGM, and in MS T2-FLAIR lesions compared to deep NAWM in MS patients.

#### 3.2.1 Group differences in microstructure, but not microvasculature, in normal-appearing tissue

Although microvascular parameters remained unchanged in MS NAWM and NAGM compartments compared with SC, significant group-differences in tissue microstructure were observed (Fig. 2). In deep NAWM, these alterations were characterized by increased MD and decreased MK, alongside elevated RD and reduced RK. In the corpus callosum NAWM, higher MK and RK were noted. Additionally, the cortical NAGM exhibited significantly lower MK in MS patients compared to SC. DKI ROI analyses by MS and SC are presented in Supplementary Table S2A.

**Fig. 2:**
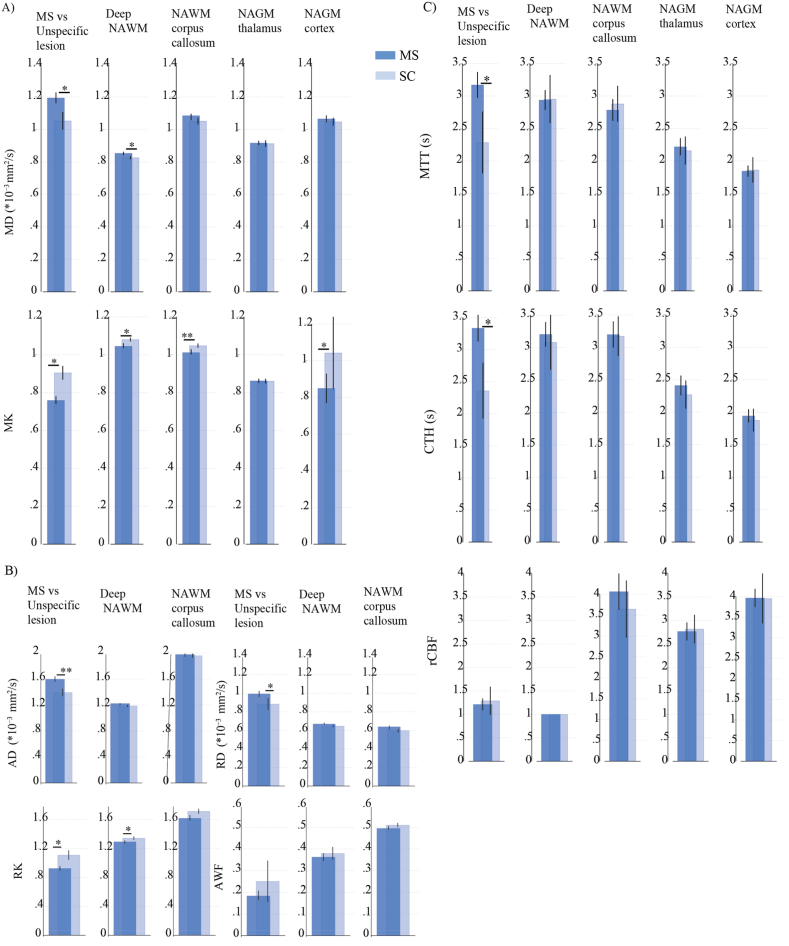
Tissue microstructure, but not microvascular perfusion, is altered in MS normal-appearing white matter. (A–B) Bar charts display mean DKI -metric values from ROI analyses of NAWM and NAGM compartments in MS patients (n = 54) and symptomatic controls (SC, n = 26). Error bars represent 95 % confidence intervals. (C) Bar charts show mean DSC-parameter values from ROI analyses of NAWM and NAGM compartments in MS patients (n = 50) and SC subjects (n = 8), with 95 % confidence intervals. Group contrasts (MS vs. unspecific) represent age- and sex-adjusted comparisons between T2-FLAIR lesions with radiological features characteristic of MS (across all subtypes) (n = 48) and unspecific T2 lesions in SC (n = 8). Group differences were assessed using multivariate regression models adjusted for age and sex. Statistical significance is indicated as *p < 0.05, **p < 0.010.1.

Normalized MS T2-FLAIR lesion load was positively correlated with MS NAWM-MD and negatively correlated with MK. There were no significant correlations between T2 lesion volume and vascular parameters (data not shown).

No significant correlations were observed between EDSS scores and structural/vascular parameters.

#### 3.2.2 Unspecific lesions resemble MS lesions microstructurally but lack vascular changes

Overlaps between microstructural degeneration and disturbed microvascular flow patterns were identified in MS T2-FLAIR lesions. Compared to unspecific T2-FLAIR lesions, MS lesions exhibited significantly increased MD and reduced MK, along with more heterogeneous microvascular flow (higher CTH) and prolonged MTT despite normal rCBF (Fig. 2). Application of the extended flow–diffusion model further revealed that MS lesions had significantly lower PtO_2_, suggesting that structural tissue damage may impair oxygen extraction through altered microvascular flow, or *vice versa*. Both unspecific and MS lesions showed increased MD and reduced MK relative to NAWM, indicating measurable tissue integrity loss. However, unlike MS lesions, unspecific lesions did not display microvascular impairment. Table 3 summarizes the overall microstructural and perfusion changes in T2-FLAIR lesions compared to NAWM control region. DKI ROI analyses by MS lesions and Unspecific lesions are presented in Supplementary Table S2B, Supplementary Material.

#### 3.2.3 Association of tissue microstructure, microvascular flow, and disease phenotype

We examined how MS disease phenotype relates to changes in tissue microstructure and microvascular flow in T2-FLAIR lesions. Significant differences were observed between patients with CIS and PPMS, as well as between RRMS and PPMS (Fig. 3). For DKI measures, but not microvascular parameters, disease-phenotype–related changes were also evident in normal-appearing tissue, with increased MD in deep NAWM, corpus callosum NAWM, thalamus NAGM, and cortical NAGM, and decreased MK in deep NAWM and corpus callosum NAWM in PPMS (Fig. S3, Supplementary Material).

**Fig. 3:**
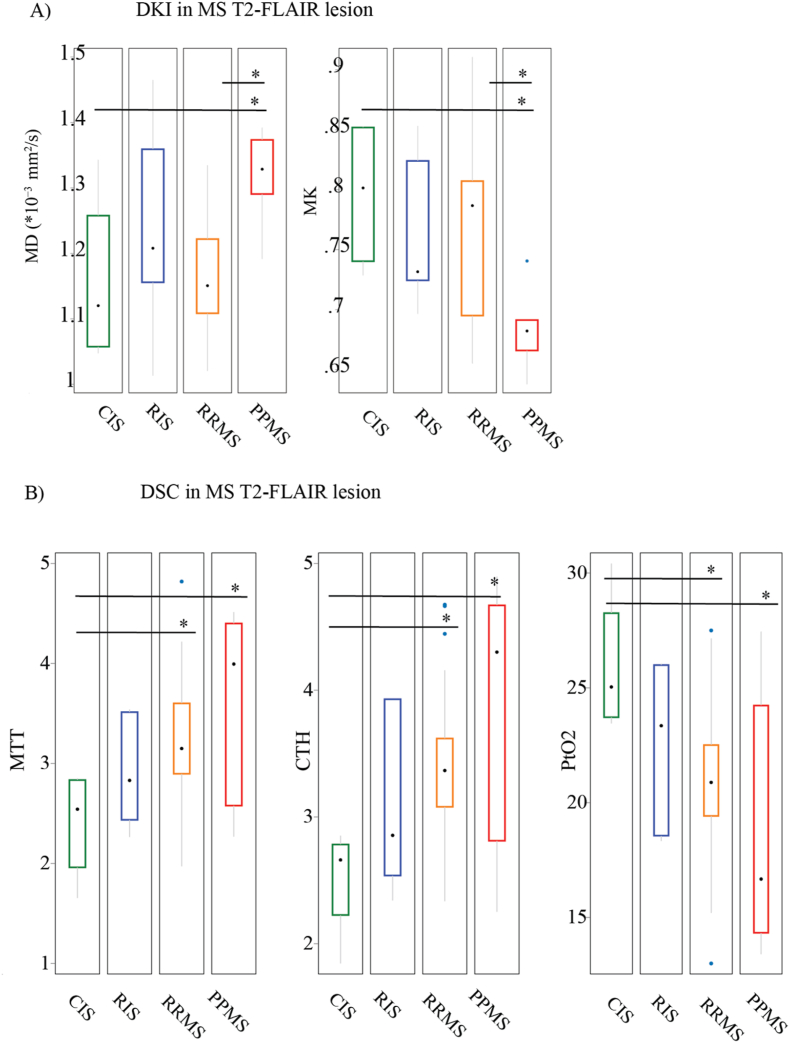
Quantitative Changes in DSC and DKI metrics in diseased tissue relate to progressive disease course. DSC and DKI are correlated in MS T2-FLAIR lesions, with quantitative changes in both metrics observed from CIS to progressive disease stage. A) Graphs depict significant differences in MD and MK estimates between CIS vs PPMS patients, and RRMS vs PPMS patients. Black dot is the median and box between the whiskers is the IQR. Multivariate regression analysis, controlling for age and sex was used to test the hypothesis of no difference between disease stages. B) Graphs depict significant differences in mean microvascular parameters estimates between CIS vs PPMS patients, and CIS vs RRMS patients. Multivariate regression, controlling for age, sex, T2-lesion volume tested the hypothesis of no difference across disease course. DKI and DSC are from MS T2-FLAIR lesion mask across different disease stage (CIS, RIS, RRMS, and PPMS; n = 4/7/30/7). Two-sided p-value less than 0.05 were considered significant.

**Fig. 4:**
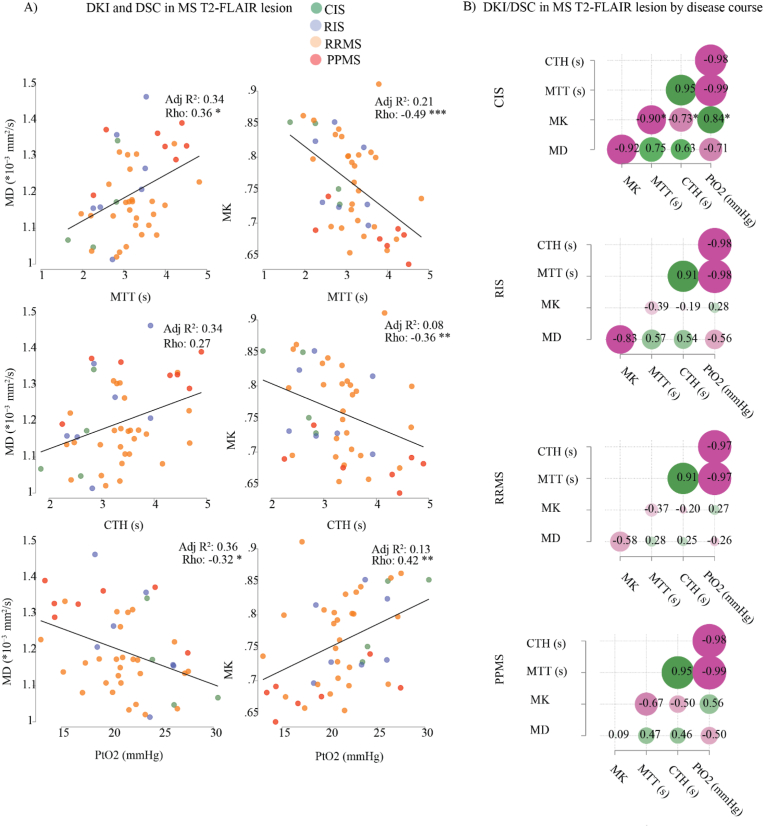
Relationships between DKI and DSC parameters in MS T2-FLAIR lesions across and stratified by disease subtype (A) Scatter plots show the relationships between DKI metrics and DSC parameters across the full MS cohort, with data points color-coded by disease subtype. The fitted regression line (STATA output) illustrates an apparent overall correlation; however, this pattern was mainly driven by between-subtype differences—with higher values in PPMS and lower values in CIS—rather than consistent within-subtype correlations. Adjusted R^2^ values indicate model fit after controlling for DSC metric, sex, and age. (B) Correlation matrices display the pairwise relationships between DKI and DSC parameters stratified by MS subtype, highlighting subtle differences in correlation strength and direction across subtypes. Circle size and color intensity represent correlation strength, with green indicating negative and pink positive associations. Spearman's rank correlation coefficient (Rho) was used. *p < 0.05; **p < 0.01; ***p < 0.001. DKI and DSC values are from MS T2-FLAIR lesion masks across disease stages (CIS, RIS, RRMS, PPMS; n = 4/7/30/7).

Across the full cohort, correlations were observed between DSC and DKI metrics, suggesting a relationship between tissue microstructure and microvascular perfusion in MS T2-FLAIR lesions (Fig. 4). Specifically, MD correlated weakly and positively with CTH and MTT, while MK correlated moderately and negatively. PtO_2_ was negatively associated with MD and positively with MK, whereas no associations were observed between rCBF and structural parameters. Stratified analyses indicated, however, that these associations were largely driven by between-group differences across MS subtypes (e.g., higher DSC and DKI values in PPMS and lower values in CIS), rather than consistent within-subtype correlations. The strongest DSC–DKI associations were found in PPMS and CIS, while these relationships were weaker or absent in RRMS and RIS (Fig. 4b). This suggests that the apparent DSC–DKI relationship reflects subtype effects rather than a generalizable continuous association across all patients.

In the multivariate regression model, we observed that age-related changes partly confounded the correlation between MD change and the DSC metric, whereas the DKI-MK measurements in MS T2-FLAIR lesions were unaffected by age (see Fig. S3 in the supplementary material).

#### 3.2.4 Correlations between microvascular and microstructural parameters across regions

**Fig. 5:**
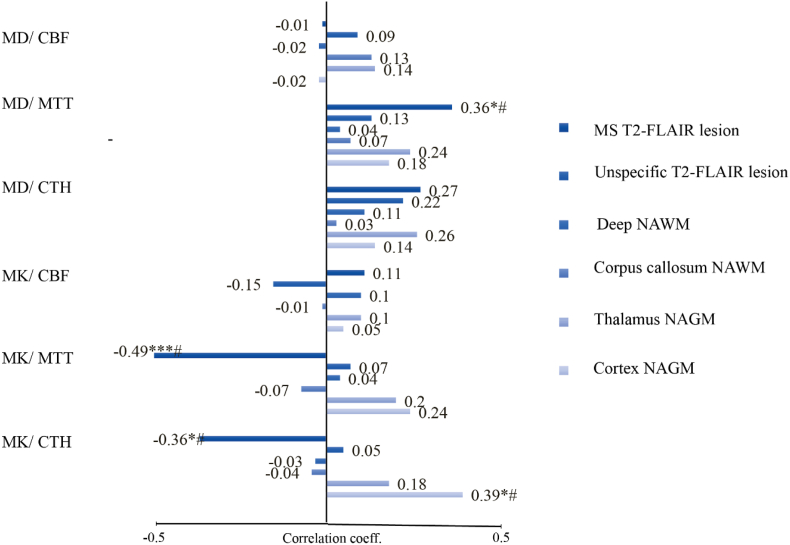
Correlation between microvascular perfusion and microstructure in MS. The relationship between microvascular perfusion and microstructure was limited to white matter regions where group differences for the modeled parameters, completely overlapped (MS T2-FLAIR lesions).Correlation bar plot display correlation coefficients between DKI - DSC metrics, derived from ROI analysis in individuals with MS (n = 50).Spearman's rank correlation coefficient (Rho) was used to assess the strength and direction of the correlations."#" indicates significant linear relationships after age adjustment.

Given that MD represents microstructural degenerative changes and the DKI and microvascular findings overlapped in MS T2-FLAIR lesions, we expected that DKI and DSC were correlated in these regions. Unexpectedly, opposing MK-CTH correlations we also found in MS patients’ Cortical NAGM (Fig. 5). Note that cortical NAGM did not show signs of altered perfusion in individuals with MS compared to SC (Fig. 2C).

#### 3.2.5 Trend toward increased diffusion restriction in enhancing T1 lesions

**Fig. 6:**
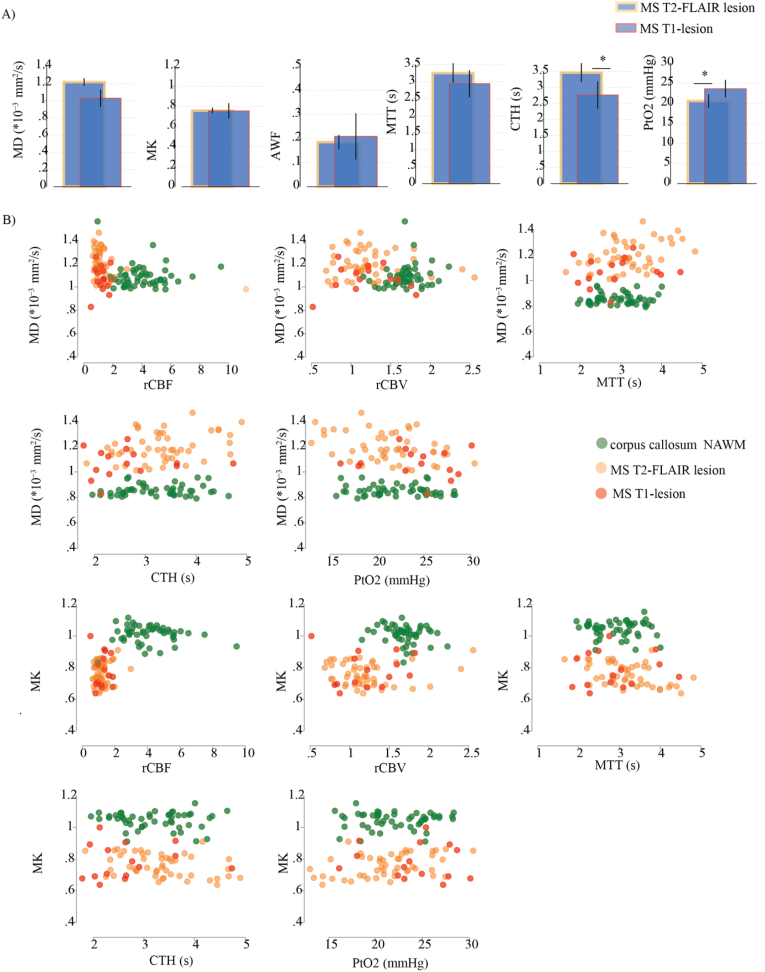
Homogenization of microvascular flows during BBB leakage is accompanied by a trend of increased diffusion restriction A) Bar charts showing differences in mean DKI metrics (MD, MK, AWF) and DSC metrics (MTT, CTH, PtO_2_) between MS T2-FLAIR in individuals without BBB leakage (n = 32) and T1 lesions in MS patients with BBB leakage (n = 16), with 95 % confidence intervals represented by vertical lines. Note, biophysically, homogenization of microvascular flows (low CTH) is noted to enhance oxygen extraction from blood supply, potentially supporting metabolic demands during inflammation and BBB damage. B) Scatter plots illustrating the relationship between DKI measurements and DSC parameters in MS lesions, with data points color-coded by contrast-enhancement, to highlight T1 lesion centered means.MS corpus callosum NAWM included as a reference. Multivariate regression analysis, adjusted for age and sex, assessed group differences. Statistical significance is denoted by *p < 0.05.

Microvascular analysis indicated relatively homogenized flow patterns, shown by low CTH in MS T1 lesions compared to MS T2-FLAIR lesions, keeping in mind that capillary flow homogenization improves oxygen extraction. This homogenization in enhancing T1 lesions was accompanied by a tendency for increased diffusion restriction (low MD) and an increase in AWF. Both MS T2-FLAIR and T1 lesions did, however, exhibit higher MD and lower MK values compared to deep NAWM (Fig. 6). Full data tables are available in Supplementary Table S3.

## 4 Discussion

Our study sheds light on the complex interplay between tissue microstructure and microvascular alterations that occur beyond the resolution of T1-and T2-weighted imaging in individuals with MS. Utilizing DKI, we identified significant group-differences in tissue microstructure in NAWM and NAGM regions, and an association between the structural changes and the progressive disease course, without co-existing change in microvascular perfusion and oxygen delivery. By combining hemodynamic measurements with the extended flow-diffusion model for oxygen transport, we observed a correlation between decreased MK—indicative of tissue integrity loss—and heterogeneous microvascular flow (high CTH) and reduction in tissue oxygenation (PtO_2_) within lesions with appearance of MS-specific demyelination (MS T2-FLAIR lesions), but no association between ‘macroscopic’ structural parameters and rCBF. Thus, the risk of compromised tissue oxygenation in MS demyelinated lesions does not appear to be due to limited blood supply, but rather to inefficient oxygen extraction from the blood (Table 1). The absence of overlapping group differences in microstructural and microvascular parameters in normal-appearing brain regions suggests that structural alterations are more readily detectable than microvascular disturbances at this stage. Instead, the altered microvascular flow patterns appear to result from the severe degenerative changes inherent to the disease process. Capillary flow disturbances are increasingly recognized in neurodegenerative and neuroinflammatory conditions and are thought to arise from age- or disease-induced structural changes in capillary walls, endothelial dysfunction, and alterations in blood rheology (Østergaard et al., 2014, 2015; Ostergaard et al., 2013; Lauer et al., 2017, 2023; Nielsen et al., 2017a). The biophysical model indicates that capillary ‘shunting’ of oxygenated blood, associated with severe capillary flow disturbances, may ultimately impair tissue oxygen metabolism, leading to hypoxic injury (Jespersen and Østergaard, 2012; Østergaard et al., 2013; Angleys et al., 2015). Thus, our findings suggesting that microvascular flow disturbances may contribute to the progression of white matter disease, highlighting the importance of longitudinal studies to further investigate the relationship between microvascular alterations and neurodegeneration, similar to those observed in Alzheimer's disease (Nielsen et al., 2017b).

Our findings are consistent with earlier pilot work linking perfusion to myelin integrity in MS lesions (Sisco et al., 2021), where changes within automated lesion ROIs were associated with altered WM microstructural integrity and weak positive correlations with perfusion parameters. They reported decreased CBF and increased MTT in lesions, consistent with hypoperfusion due to reduced blood flow. By comparison, in our study MS T2-FLAIR lesions showed increased MTT and CTH despite preserved CBF, suggesting that heterogeneous flow reflects alterations in microvascular function or patency (Fig. 2). We therefore propose that microvascular flow disturbances should be factored in when studying vascular–tissue interactions in MS. Also note that while several studies have reported hypoperfusion in NAWM and NAGM, particularly in progressive MS((Hojjat et al., 2016; Adhya et al., 2006; Varga et al., 2009; Ge et al., 2005; Rashid et al., 2004)), such findings vary with disease stage, treatment status, and methodological differences. Our results in MS NAWM align with previous align with prior DSC investigations on healthy subjects (Nielsen et al., 2017b), suggesting that MS-related hypoperfusion may not be universal. Current studies have yet to confirm whether hypoperfusion precedes neurodegeneration.

### 4.1 Disease-phenotype and age-associated brain changes

In exploring disease- and age-related changes, both DKI and DSC metrics revealed alterations linked to MS phenotype. Among these, DKI-MK emerged as the most sensitive indicator of diffusion abnormalities in both WM and GM, reflecting altered tissue complexity. While previous studies have demonstrated the utility of DKI in detecting diffuse brain damage in MS (Bester et al., 2015; Thaler et al., 2021; de Kouchkovsky et al., 2016; Yoshida et al., 2013; Li et al., 2018; Luo et al., 2024; Zhu et al., 2022), no single metric has consistently proven superior across research. In our dataset, both DKI-MD and DKI-MK, together with microvascular parameters (CTH and MTT), were associated with the progressive disease course, with changes in NAWM, NAGM, and MS lesions being most pronounced in PPMS (Fig. 3). Similarly, the coupling between DSC and DKI metrics was most evident when comparing across different MS subtypes, and appeared to reflect differences between subtypes (e.g., PPMS vs CIS) rather than consistent correlations within each subtype (Fig. 4). Together, these findings suggest that tissue damage and vascular dysfunction may be more important in certain disease stages rather than uniform across MS.

Although all diffusion parameters correlated with age in highly vascularized GM, only DKI-MD showed significant overlap with age-related effects in MS T2-FLAIR lesions (Fig. S3 in the supplementary material). Kurtosis-based correction by DKI has previously been shown to enhance the detection of age-related changes compared to traditional DTI metrics (Taha et al., 2022). Distinguishing between disease-related and age-related changes is particularly challenging in progressive MS, where both processes may converge. Evidence suggests that accelerated aging is an inherent feature of MS pathophysiology (Giovannoni et al., 2020). Shared mechanisms, including iron accumulation (Haider et al., 2014), microglia activation (Lassmann, 2014), mitochondrial damage (Witte et al., 2014; Chandler et al., 2023), oxidative stress (Trapp and Stys, 2009; Mahad et al., 2015; Haider et al., 2011; Lu et al., 2000), and endothelial dysfunction (Cashion et al., 2023; Cramer et al., 2014; Iadecola, 2017), contribute to both MS neurodegeneration and aging. Impaired cerebrovascular reactivity is likewise observed with aging and in progressive MS (Vestergaard et al., 2022), possibly reflecting exhausted vascular reserve or vascular inflammation. Together, these mechanisms may render brain tissue and vasculature more vulnerable to aging, thereby promoting progression in MS (Amezcua, 2022; Giovannoni et al., 2020; Graves et al., 2023). Further investigation is needed to explore the potential of targeting microvascular flow disturbances as a therapeutic strategy for progressive MS.

### 4.2 DKI metric interpretation

Our observations of increased MD and decreased MK in MS NAWM compared to SC WM, and in MS lesions compared to MS patient NAWM, align with previous research findings employing DTI or DKI-derived metrics (van der Weijden et al., 2022; Werring et al., 1999; Thaler et al., 2021; de Kouchkovsky et al., 2016; Zhu et al., 2022). Together, findings indicate an overall loss of structural barriers and tissue integrity in the MS-affected brain. Additionally, DKI-derived RD and AD in our study distinguished demyelinated MS T2-FLAIR lesions from deep NAWM and focal unspecific T2-FLAIR lesions. Prior studies in rodent models have suggested that RD is more sensitive to hypomyelination than AD(43, 45), though findings vary (Guglielmetti et al., 2016). Variations across studies in terms of their sensitivity to pathological changes may be due to differing methodologies, particularly the failure to include kurtosis in diffusion weighted imaging studies. Additionally, high AD values have been associated not only with longstanding demyelination (Guglielmetti et al., 2016; Falangola et al., 2014) but also with astrogliosis (Guglielmetti et al., 2016; Falangola et al., 2014; Wang et al., 2009).

Astrogliosis, triggered by inflammatory and hypoxic stimuli, plays a significant role in MS lesion formation, driving the transition from acute inflammatory injury to a state of sclerotic scarring (Ponath et al., 2018). While some oxidative stress may result from mitochondrial damage secondary to chronic neuroinflammation (Trapp and Stys, 2009; Chandler et al., 2023; Mahad et al., 2015), our findings suggest that impaired oxygen extraction due to microvascular flow disturbances in MS lesions also contributes to inefficient energy production, thereby facilitating the pro-inflammatory process. Notably, Dalby et al., using DTI tractography with DSC in non-inflammatory white matter disease, linked flow disturbances in the affected fiber tracts to disease burden (Dalby et al., 2019). This methodological approach could be further explored to enhance our understanding of the sources of elevated oxidative stress and structural damage in the NAWM of individuals with MS.

### 4.3 Differentiating MS lesions from unspecific lesion using DKI and DSC metrics

While MS lesions typically possess distinct radiological characteristics recognizable to experienced MS specialists or neuroradiologists, distinguishing them from nonspecific white matter hyperintensities using conventional T1-and T2-weighted MRI can be challenging (Kaisey et al., 2019; Solomon and Weinshenker, 2013; Filippi et al., 2019) Nonspecific lesions, commonly incidental findings, are frequently associated with aging, migraines, or mild chronic ischemia, and their origin is presumed to be microvascular (Wardlaw et al., 2015; Medrano et al., 2012)

In the current study, Unspecific lesions exhibited measurable loss of tissue integrity compared to NAWM (Fig. 2), however, they did not demonstrate the microvascular impairment that was characteristic of MS-specific lesions. We recognize that differences in lesion distribution between MS-specific and Unspecific lesions could reflect varying relationships with underlying vascular territories. Future studies may benefit from transposing patient lesion maps onto DSC maps from age- and sex-matched healthy controls, thereby enabling precise comparative analyses of identical anatomical regions in patients and controls. We also acknowledge that that some lesions categorized as “unspecific” may still represent MS pathology. Therefore, not incorporating advanced MRI sequences such as T2* and susceptibility-weighted imaging (SWI) may have limited our lesion classification accuracy, representing a methodological limitation of this study.

The technical limitations of both DKI and DSC MRI, particularly their differing spatial resolutions, must be considered. The variation in voxel sizes between T2-FLAIR (1 mm), DKI (2 mm), and DSC (3 mm) introduces partial volume effects (PVE) when averaging parameters within ROIs. This issue is most pronounced in small lesions, where a larger proportion of voxels may contain both lesion and adjacent normal tissue, thereby reducing lesion–NAWM contrast. In summary, PVE probably made the small (mostly non-specific) lesions appear more similar to NAWM, but the relative contribution of PVE versus biological factors to the observed group differences remains uncertain. Because analysis was performed using aggregate lesion masks per participant and not at the individual lesion level, analysis of sensitivity and propagation of error analysis was not performed but represents an important avenue for future investigations.

To mitigate PVE, we chose to manually delineate lesion, and we placed the WM ROIs in the deep NAWM and the central middle of the corpus callosum. Despite these efforts, PVE may have influenced the MD results, particularly in the corpus callosum, where unexpectedly high MD values were observed. The omission of whole-brain NAWM and automated lesion segmentation masks was primarily due to the challenges in automatically detecting MS-specific demyelination and excluding artifacts from WM analysis, given their overlapping intensity with normal tissue.

### 4.4 Microstructural and hemodynamic features related to contrast enhancement

Integrating our findings with DSC measurements, we observed that homogenization of microvascular flows (low CTH) in active T1 lesions was associated with a trend toward increased diffusion restriction and elevated AWF. Keeping in mind that flow homogenization is proposed as a biophysical marker of active hemodynamic responses that enhance net oxygen extraction (Table 1), potentially serving to meet the metabolic demands of ongoing inflammatory processes. DTI-derived metrics have been reported to be more reliable in cases with minimal edema and inflammation (Winklewski et al., 2018). Similarly, the ability of DKI to differentiate between active enhancing T1 lesions and chronic MS T2-FLAIR lesions remains unclear (Thaler et al., 2021; Zhu et al., 2022). This ambiguity may result from overlapping pathological processes, such as axonal swelling, edema, hypercellularity, and myelin breakdown, which are associated with BBB leakage. In isolation, some of these processes cause increases in DKI metrics while others cause decreases. When several such processes coexist, the overall effect on DKI metrics may, therefore, be very subtle, erratic, or vary from patient to patient (Werring et al., 1999; Hannoun et al., 2015). One thing to note, though, is that DKI sensitivity is known to be affected by the choice of b-value and that the optimal b-value is different in WM and GM (Chuhutin et al., 2017). Therefore, optimized protocols for DKI in MS lesions might enable better differentiation between lesion types and states. Dedicated protocols distinguishing active contrast-enhancing lesions and chronic black holes could further help disentangle their distinct microstructural and microvascular profiles, which could be obscured when non-enhancing lesions are analyzed collectively.

### 4.5 Limitations

A key limitation of our study is its cross-sectional design. Although our findings did not reveal evidence of microvascular flow disturbances within NAWM, we cannot exclude the possibility that inflammation-induced microvascular changes contribute to subsequent neurodegeneration. Moreover, recruiting patients from the entire spectrum of MS resulted in a heterogeneous cohort. Given the considerable variability within the dataset, a larger sample size would have increased the statistical power. These considerations emphasize the importance of conducting longitudinal studies with homogeneous cohorts to more fully elucidate the role of vascular health in the progression and clinical manifestations of MS. As this was an exploratory study, we did not apply formal multiple comparison correction. Because vascular impairment must be interpreted from a panel of interrelated parameters (e.g., CBF, CBV, MTT, CTH, PtO_2_), strict corrections assuming independence would risk obscuring biologically meaningful effects. Instead, we emphasized converging patterns across interrelated measures, and results should therefore be interpreted with appropriate caution.

Despite limitations, our study's strengths include a well-characterized non-diseased control group with normal clinical and radiological findings. We consider the SC group a clinically relevant reference population that reflects the diagnostic challenges of everyday practice, though not “healthy controls” in the strict sense. We acknowledge that a small subset may later convert to MS or another inflammatory disease. Two SC participants had oligoclonal bands, suggesting possible subclinical or future risk of CNS involvement, yet without a confirmed diagnosis within two years. Future studies should therefore recruit both SC and healthy volunteers to better distinguish abnormalities specifically associated with MS pathology.

## 5 Conclusion

By correlating DKI/DSC findings and integrating the extended flow-diffusion model of oxygen transport, we identified a relationship between tissue integrity loss and microvascular disturbances in MS-specific demyelinating lesions. This suggests that both indices reflect critical aspects of the neurodegenerative process in MS. The tissue microstructure was altered in MS NAWM and NAGM regions without accompanying changes in microvascular perfusion or oxygen delivery, suggesting that altered microvascular flow patterns in damaged tissue may reflect the severe degenerative changes inherent to the disease. These observations indicate that microvascular disturbances, possibly in conjunction with degenerative changes, may contribute to the progression/acceleration of WM disease, warranting further longitudinal research from a treatment perspective.

## CRediT authorship contribution statement

**Linda Sundvall:** Writing – review & editing, Writing – original draft, Visualization, Investigation, Formal analysis. **Mikkelsen Irene Klærke:** Writing – review & editing, Validation, Supervision, Software, Methodology, Investigation. **Brian Hansen:** Writing – review & editing, Validation, Supervision, Methodology, Conceptualization. **Simon Fristed Eskildsen:** Writing – review & editing, Validation, Supervision, Methodology. **Mette Madsen Hjørringgaard:** Writing – review & editing, Validation, Investigation. **Mikkel Karl Emil Nygaard:** Writing – review & editing, Validation, Software, Methodology. **Peter Vestergaard Rasmussen:** Writing – review & editing, Resources. **Thor Petersen:** Writing – review & editing, Supervision, Resources, Conceptualization. **Leif Østergaard:** Writing – review & editing, Supervision, Resources, Methodology, Conceptualization.

## Grant support

This research received financial support from the Danish Multiple Sclerosis Society, Jascha Fonden, Hestehandler Ole Jakobsens Mindelegat, Novartis Healthcare, Dagmar Marshalls Fond, and Augustinus Fonden. LØ is supported by The Lundbeck Foundation (Grant no. R310-2018-3455). The sponsors provided funding exclusively for conducting the research, with no involvement in the article's preparation, study design, data collection, analysis, interpretation, or the decision to submit for publication.

All authors have given their final approval of the present version to be published.

## Declaration of competing interest

The authors declare the following financial interests/personal relationships which may be considered as potential competing interests:Linda Sundvall reports financial support was provided by Danish Multiple Sclerosis Society. Linda Sundvall reports financial support was provided by Augustinus Foundation. Linda Sundvall reports financial support was provided by Dagmar Marshall Fund. Linda Sundvall reports financial support was provided by Horse Sales Ole Jakobsens Memorial Scholarship. Linda Sundvall reports financial support was provided by Jascha Foundation. Leif Ostergaard reports financial support was provided by Lundbeck Foundation. Leif ostergaard reports a relationship with Cercare Medical that includes: board membership. If there are other authors, they declare that they have no known competing financial interests or personal relationships that could have appeared to influence the work reported in this paper.

## Figures and Tables

**figs1:**
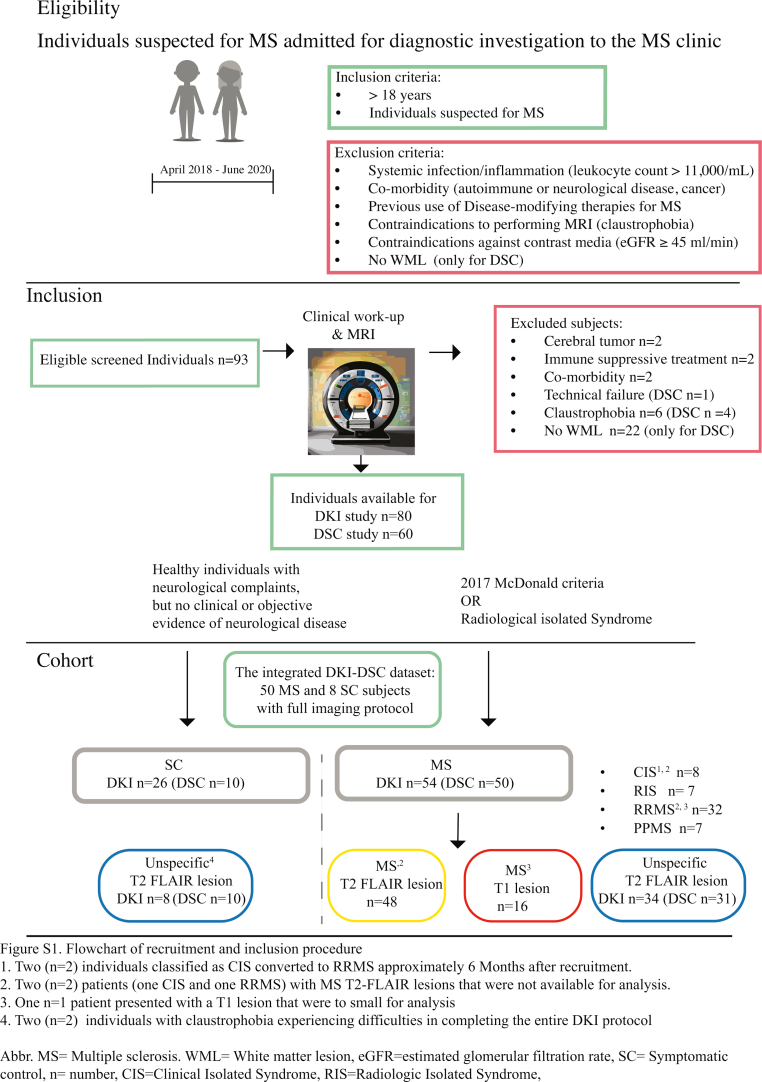


**figs2:**
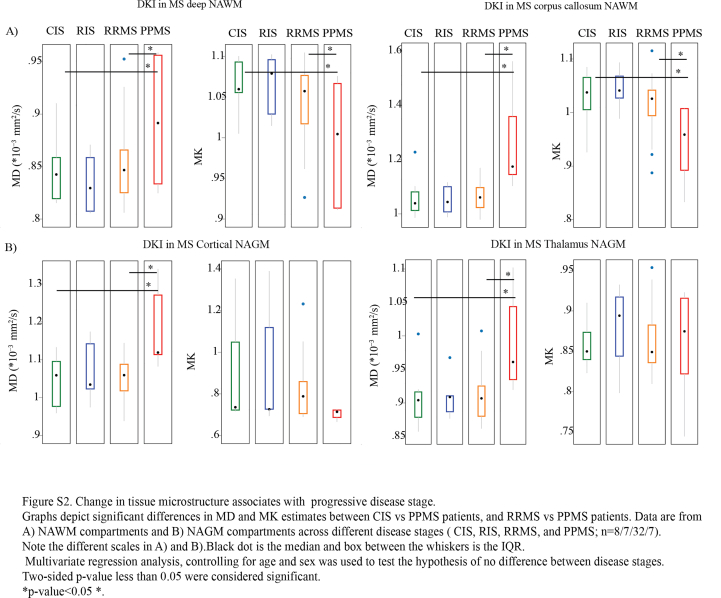


**figs3:**
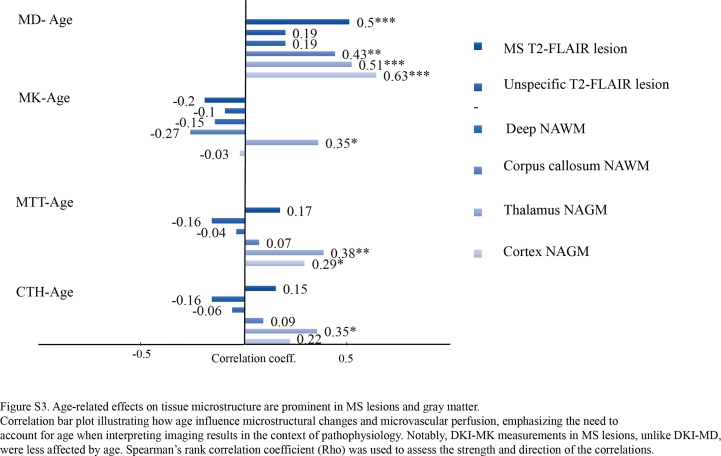


## Data Availability

The authors do not have permission to share data.
