# Supplementary material for: Neurodegeneration and energy depletion in MS: Links between tissue integrity loss and microvascular changes in white matter disease
Source: Neuroimage Rep. 2025 Dec 10;6(1):100309. doi: 10.1016/j.ynirp.2025.100309 (PMC12753510; doi:10.1016/j.ynirp.2025.100309)
Supplement: Multimedia component 1 [file mmc1.docx]

## Supplementary material methods

# Supplementary methods

1. MRI protocol
2. MR post-processing
   - - 1. MRI protocol

The MRI protocol included three‐dimensional (3D) T2-weighted fluid-attenuated inversion recovery (T2-FLAIR) images and post-contrast 3D T1-weighted (T1) images for lesion outlining, Diffusion kurtosis imaging (DKI) sequence for microstructural assessment and a gradient-echo dynamic susceptibility contrast (DSC) scan for perfusion and brain oxygenation assessment as well as pre-contrast 3D T1 magnetization‐prepared 2 rapid gradient‐echo (MP2RAGE) for detailed structural assessment and tissue segmentation. All MR images were acquired on the Siemens 3T Prisma scanner;

Pre-contrast sagittal 3D T2-FLAIR images for T2-FLAIR lesion outlining were acquired in 176 slices, TE = 388 ms, TI= 1800 ms, TR = 5000 ms, FA=120°, no slice gap, 1 × 1 × 1 mm^3^ voxel.

Pre-contrast sagittal 3D T1 MP2RAGE images for tissue segmentation into gray matter (GM), white matter (WM), cerebrospinal fluid (CSF) and white matter lesion (WML) were acquired with 192 slices, TE = 3.47 ms, TI_1_= 500 ms, FA_1_=4°, TI_2_= 2900 ms, FA_1_=7°, TR = 6500 ms, no slice gap, 0.9 × 0.9 × 0.9 mm^3^ voxel.

DKI images were acquired with 72 axial slices, TE = 63ms, TR = 2260ms, no slice gap, 2 × 2 × 2 mm^3^ voxel in a 100x100x72 matrix. We acquired *b*-values between 0 and 2500 s/mm^2^, along 201 distinct directions for robust estimation of DKI parameters. The diffusion weightings were distributed on the b-value shells as follows where the number in parenthesis is directions or repetitions: b=0 s/mm^2^ (10 reps), b=700 s/mm^2^ (15), b=1000 s/mm^2^ (30), b=1200 s/mm^2^ (21), b=1500 s/mm^2^ (60), b=2500 s/mm^2^ (75). One volume without diffusion weighting was acquired in the PA direction for eddy-current correction.

DSC perfusion sequence was a gradient echo EPI sequence acquired with 42 axial slices, TE = 32 ms, TR = 0.8s, flip angle 45 or 53 degrees, no slice gap, transverse slice orientation, 3.0 × 3.0 × 3.0 mm^3^ voxel in 64x64 matrix. The paramagnetic contrast agent Gadovist (1M, Bayer) was administered with 0.1mmol per kg body weight with a power injector at an injection rate of 5ml/s and followed by a 30 ml saline flush. Injection commenced 15 seconds after DSC imaging onset to provide a baseline before contrast arrival to the brain, and bolus duration was standardized to 60 seconds after contrast arrival.

Post-contrast sagittal 3D T1 MPRAGE was acquired to outline enhanced T1 lesions. The imaging parameters were 176 slices, TE = 2.52 ms, TI= 900 ms, TR = 1900 ms, no slice gap,1 × 1 × 1 mm^3^ voxel, 9° flip angle.

- - - 1. MR post-processing

1. Tissue segmentation: T_1_w images were processed using a fully automatic pipeline(1). Images were denoised(2), registered to MNI space using a 12-parameter affine transformation(3) and skull stripped(4). Regional brain volumes (i.e., hippocampus, caudate nucleus, putamen, globus pallidus, thalamus, and corpus callosum) were segmented using a patch-based label-fusion method(5), applying manually crafted training data from the same scanner, which has been shown to improve accuracy compared to other methods(6). Tissue segmentation was performed to provide binary structural masks of GM, WM, and CSF using an automated neuronal network classification (7) combined with tissue priors non-linearly warped from MNI space to the individual images(8). The mid-sagittal segmentation of the corpus callosum was dilated in the lateral direction to achieve a thickness of 3 mm. Additionally, axial T1 images in standard space were employed to manually define frontal and parietal deep WM regions in each hemisphere, and warped to each subject’s T1 images. These were designated as the deep NAWM region for the subsequent region of interest analysis and were also used for normalization of CBF and CBV maps in DSC.
2. T2-FLAIR lesion masks and enhancing T1-lesion masks were manually drawn on sagittal 3D T2-FLAIR and post-contrast sagittal 3D T1 images, respectively. Additionally, an auto-generated non-modified WML mask was segmented by the lesion growth algorithm(9) as implemented in the LST version toolbox 2.0.15 (www.statisticalmodelling.de/lst.html) implemented in Statistical Parametric Mapping toolbox (SPM12) running in MATLAB R2016b (MathWorks, Natick, MA, USA).
3. DKI data processing and parameter estimation: First, the diffusion weighted volumes were denoised(10), corrected for Gibbs ringing artifacts(11), followed by standard corrections for susceptibility-induced distortions, motion(12, 13), eddy-current-induced distortions, and bias field correction (using FSL’s bet, top up and eddy)(14). The corrected diffusion data were subsequently used to estimate the DKI parameters (MD, AD, RD, MK, RK) using in-house matlab (Mathworks, USA) scripts for DKI estimation using outlier rejection (53).
4. Generation of parametric DSC perfusion maps: All dynamically imaged volumes were truncated from bolus arrival until 60 seconds post-bolus to standardise bolus duration and minimise bias from individual variations in arrival times. Vascular comorbidities can alter hemodynamic properties, affecting bolus arrival shape and amplitude and potentially introducing bias into perfusion parameter estimation. To reduce these effects, the arterial input function (AIF) was automatically selected (high peak quick washout criteria) in an image slice confined to the region supplied by the middle cerebral artery. The AIF for each imaging session was visually inspected to ensure that the selected voxel was suitable for AIF. The generation of parametric perfusion maps was adjusted for variations in contrast agent arrival by deconvolving the arterial input function (AIF). This ensured that differences in distance between the regions or lesion masks examined and the AIF would not confound the resulting parametric perfusion maps. Slice timing correction was performed, and concentration was calculated using the exponential relation between MR signal changes in the relaxation rate that occurs as the contrast agent passes the vasculature. The contrast curve representing the arterial supply (arterial input function) was automatically detected among voxels with the narrow and early arrival of the contrast agent and with a fast initial increase in area under the concentration curve. Spatially, the search was confined to the region supplied by the middle cerebral artery. Large vessels and CSF were excluded, and the images were smoothed using a Gaussian 3x3 voxels in-plane filter.

No temporal smoothing was performed. The tissue concentration curves were corrected for the dispersion of contrast already present in the arterial supply in a deconvolution process. The tissue curves after correction describe the remaining concentration in the capillary web had the bolus been injected instantly into the tissue. The deconvolution strategy was based on a parametric method by Mouridsen et al. and directly relates the residue function R(t) to the distribution of vascular transit times, h(t), and hence to CTH. This residue function is modeled by a family of gamma variate functions(15).Specifically, the model includes the delay between supplying artery and tissue curves, the CBF, and two model parameters, alpha and beta, describing the shape of the corrected tissue curves. The parameters were fitted using an expectation-maximization fitting algorithm. The starting guess of the parameters was estimated using singular value decomposition. MTT and CTH, being the mean and standard deviation of the distribution of capillary transit times, are given by the gamma variate function. The deconvolution method yields CBF×R(t), the height of which is CBF, because R(t) quantifies the fraction of contrast media retained in the microcirculation. CBV corresponds to the area under CBF×R(t) curve, following the central volume theorem (CBV=CBF*MTT). Using MTT and CTH estimates, a biophysical model by Jespersen and Oestergaard was used to calculate corresponding tissue oxygen pressure (ptO2)(16).

Due to technical and physiological variations in absolute MRI DSC perfusion measures, CBF and CBV estimates are not reliably comparable across individuals and are often reported in arbitrary units or normalized to a reference region within the same subject. Therefore, we normalized CBV and CBF measures in lesions and normal-appearing compartments (corpus callosum NAWM, thalamus NAGM, cortical NAGM) to deep NAWM within the same subject.

1. Co-registration: Manually drawn lesion masks, auto-generated structural masks, and structural 3D T1 MP2RAGE and 3D T2-FLAIR images were co-registered and resliced to the average DKI b = 0 images and mean DSC image volume using SPM12.
2. Region-of-interest (ROI) analysis. ROI analysis involved extracting DKI and DSC metrics from both manually drawn lesion masks and auto-generated masks of normal-appearing white matter (NAWM) and normal-appearing gray matter (NAGM) in specific structures (corpus callosum, deep WM, thalamus, and cortex). All ROIs were excluded from CSF and large vessels prior to ROI extraction. In ROI analysis for NAWM/ NAGM compartments, the WM/GM mask was used as the inclusion and auto-generated WML masks, and the manually drawn lesion masks were used as exclusion masks. In ROI analysis for lesions, MS T2-FLAIR lesions containing any overlap with T1-lesion masks were excluded.

References

1. Aubert-Broche B, Fonov VS, García-Lorenzo D, Mouiha A, Guizard N, Coupé P, et al. A new method for structural volume analysis of longitudinal brain MRI data and its application in studying the growth trajectories of anatomical brain structures in childhood. Neuroimage. 2013;82:393-402.

2. Coupe P, Yger P, Prima S, Hellier P, Kervrann C, Barillot C. An optimized blockwise nonlocal means denoising filter for 3-D magnetic resonance images. IEEE Trans Med Imaging. 2008;27(4):425-41.

3. Collins DL, Neelin P, Peters TM, Evans AC. Automatic 3D intersubject registration of MR volumetric data in standardized Talairach space. J Comput Assist Tomogr. 1994;18(2):192-205.

4. Eskildsen SF, Coupé P, Fonov V, Manjón JV, Leung KK, Guizard N, et al. BEaST: brain extraction based on nonlocal segmentation technique. Neuroimage. 2012;59(3):2362-73.

5. Coupé P, Manjón JV, Fonov V, Pruessner J, Robles M, Collins DL. Patch-based segmentation using expert priors: application to hippocampus and ventricle segmentation. Neuroimage. 2011;54(2):940-54.

6. Naess-Schmidt E, Tietze A, Blicher JU, Petersen M, Mikkelsen IK, Coupe P, et al. Automatic thalamus and hippocampus segmentation from MP2RAGE: comparison of publicly available methods and implications for DTI quantification. Int J Comput Assist Radiol Surg. 2016;11(11):1979-91.

7. Zijdenbos AP, Forghani R, Evans AC. Automatic "pipeline" analysis of 3-D MRI data for clinical trials: application to multiple sclerosis. IEEE Trans Med Imaging. 2002;21(10):1280-91.

8. Collins DL, Evans AC. Animal: Validation and Applications of Nonlinear Registration-Based Segmentation. International Journal of Pattern Recognition and Artificial Intelligence. 1997;11:1271-94.

9. Schmidt P, Gaser C, Arsic M, Buck D, Förschler A, Berthele A, et al. An automated tool for detection of FLAIR-hyperintense white-matter lesions in Multiple Sclerosis. Neuroimage. 2012;59(4):3774-83.

10. Veraart J, Novikov DS, Christiaens D, Ades-Aron B, Sijbers J, Fieremans E. Denoising of diffusion MRI using random matrix theory. Neuroimage. 2016;142:394-406.

11. Kellner E, Dhital B, Kiselev VG, Reisert M. Gibbs-ringing artifact removal based on local subvoxel-shifts. Magn Reson Med. 2016;76(5):1574-81.

12. Andersson JL, Skare S, Ashburner J. How to correct susceptibility distortions in spin-echo echo-planar images: application to diffusion tensor imaging. Neuroimage. 2003;20(2):870-88.

13. Smith SM, Jenkinson M, Woolrich MW, Beckmann CF, Behrens TE, Johansen-Berg H, et al. Advances in functional and structural MR image analysis and implementation as FSL. Neuroimage. 2004;23 Suppl 1:S208-19.

14. Andersson JLR, Sotiropoulos SN. An integrated approach to correction for off-resonance effects and subject movement in diffusion MR imaging. Neuroimage. 2016;125:1063-78.

15. Mouridsen K, Hansen MB, Østergaard L, Jespersen SN. Reliable estimation of capillary transit time distributions using DSC-MRI. J Cereb Blood Flow Metab. 2014;34(9):1511-21.

16. Jespersen SN, Østergaard L. The roles of cerebral blood flow, capillary transit time heterogeneity, and oxygen tension in brain oxygenation and metabolism. J Cereb Blood Flow Metab. 2012;32(2):264-77.
